# The Examination at Apothecaries' Hall

**Published:** 1834-01-01

**Authors:** 


### II. THE EXAMINATION AT APOTHECARIES' HALL.

As some men are born great, and others achieve greatness, so others, says the old saw, have greatness thrust upon them. The Company of Apothecaries are an example of this freak of fortune: by a legislatorial joke, (as when Sancho was made Governor of Barataria,) a knot of traders has been elevated into the most powerful body in the medical profession; and, if the purple robe of authority sits but awkwardly on the dealers in mustard and arrow-root, the fault is with those who insisted on the strange disguise, and determined on creating lords of misrule. We justly murmur at authority when it is exercised by those who, having neither birth, nor education, nor splendid talents to boast of, can appeal neither to the instincts of nature, nor to the conventional usages of society; and we suspect that no man with a diploma in his pocket (though it were only a diploma of St. Andrew's), ever went up to the examination in Blackfriars' without a deep feeling of humiliation. Yet, though the examination may be bad, it does not follow that everything which can be said against it is good: and we must confess that the writer of the following observations has very much the air of a man who has been more than half flayed: like a modern Marsyas he complains that the examination of these little gods of physic is both harassing and superficial. We think it rather a wild theory too, that people at this end of the world preferred him "as an educated Scotsman to any of their own countrymen;" but we must recollect that these sorenesses first saw the light in Glasgow; and, as Aristotle says, " it does not go against the grain to praise the Athenians at Athens."

The Apothecaries' Company of London the other day, in their memorandum or remonstrance to Lord Melbourne, mentioned their having frequently rejected the Scottish candidates for their diploma, even when these gentlemen possessed the highest Scottish medical honours; and they wish the world to believe from this, that the diploma of the worshipful company is a higher evidence of medical merit, than any diploma or degree from Scotland, as is evinced by gentlemen possessing all the honorary titles and qualifications to boot of Edinburgh, Glasgow, St. Andrew's, or Aberdeen, being unable to pass *their* ordeal. All this is very strange; but there are one or two things behind the curtain which the world must know, to be able correctly to form an opinion of these worshipful worthies. The English public are perfectly aware of the superiority of the Scottish educated practitioners. This is no vain boast or hearsay; the writer of this has been told it repeatedly by individuals educated and uneducated, who were utter strangers to him, and who seemed glad of the opportunity of telling him they could trust in him as an educated Scotsman, in preference to any of their own countrymen,—of this predilection the apothecaries are perfectly aware, and their cue is to keep as many out as they can, thus reserving to themselves as large a share of the good things of the profession as possible. There are a variety of ways for attaining this. The five years apprenticeship was a dextrous and a pretty successful one—the minute and often silly differences between the pharmacopœias another; if these do not succeed, they have the ungentlemanly one of browbeating the candidates during examination, in such a manner that few nervous men are able to stand it. Before the examination commences, they usually contrive to make out if the candidate has passed any other college; if he has, of course they delight in rejecting him if they can, because by this means they not only keep up still further the monopoly of practice, but afford themselves another instance to prove, according to them, that a man may have obtained the highest honours of those schools most celebrated for their alumni, and yet be altogether unable to pass the examination required by the worshipful company of apothecaries. I knew an instance where a gentleman who did not know them so well as I do, signed the Latin translation he had to make, and added "M.D. of Edinburgh, &c.;" the consequence was, whenever he entered the room, he was *ordered*, in a surly gruff voice, to "write that over anew, sir—we don't want any of your M.D.'s—it will not serve your purpose here, sir,"—and neither it did, for they rejected him. The apothecaries boast that none of their examining board teach, consequently can have no interest in rejecting or passing the candidates; but it appears to me that there is as little objection to a teacher as to a practitioner. As practitioners, the London apothecaries are objectionable examinators, inasmuch as they are tempted to keep as many out of the way of competition as possible, which they easily hitherto could accomplish by unjust bye-laws, and equally unjust examinations.

If the reader will turn to No. 495 of the Lancet, for 23d of February, 1833, he will get a specimen of the examination customary at the hall in Bridge street, London, and after he has read it he will easily understand by what *laudable* means the Scottish applicants are rejected, and with what decency the apothecaries can boast of having rejected so many from this side of the Tweed. The examination alluded to was that of the writer of this article; and, though they did their best to throw him out, they did not succeed.

Their apprentice system is another of their boasted means of securing a fine medical education in the rising generation of medical aspirants. It has been my lot to be thrown not a little in the way of these young gentlemen, and I can safely say that it is of almost no value to the apprentice whatever, but of considerable importance to the master, a handsome fee in the first place of from 150*l.* to 500*l.*, and gratuitous services during five years, have hitherto been pretty reasonable sources of emolument. All the disagreeable drudgery part of practice is done by the apprentice, and at the end of five years he goes to town with just as much information as could easily be acquired by six months' assiduous attention in the shop of any respectable practitioner.

Many of them go out as assistants at the end of their apprenticeship, and if in town, and their master kind, contrive to attend a class or two—and to these young men often are cases of considerable importance entrusted, in the absence of the principal. One very gentlemanly young man was assistant to a friend of mine in good practice in the west end of London, and I never recollect being so much astonished as on discovering the ignorance of my young friend. He had been five years an apprentice at Exeter, and one year at the classes in London, and knew nothing—absolutely nothing,—to be able merely to make a pill or mix a powder, I call nothing. A man surely does not require five years to get up such splendid acquirements as these, and yet this gentleman, of some three and twenty years of age, and six years' standing in the profession, had not information enough to enable him to discover an inflamed gum, or any one of the commonest "ills that flesh is heir to," and when pointed out to him, had no idea of how it was to be remedied. So much for the value of the five years' apprenticeship.—*Glasgow Medical Journal*, Oct. 1833.

Some of the mistakes the writer makes in the course of his article are so gross, that we are amazed at the facile good-humour of the Southrons, who preferred him to any of their countrymen; and our only reason for not calling him a half-informed person is, that his information has by no means reached so respectable a fraction. Thus he asks, "how are men, who have attended a short course or so upon such a subject as chemistry, to be expected to answer questions regarding the atomic theory—a very common subject at the hall?" How obvious is the answer! The student who desires to learn chemistry, will not be content with what he hears in the lecture-room, but he will read and perform experiments for himself. The author concludes with the threadbare objection to Cambridge and Oxford, that they form deep scholars, but that scholarship is not wanted in the medical profession.

Answer.—It *is* wanted for the heads of the medical profession, unless we wish the practitioners of physic to be ranked with mechanics and artizans; the most useful persons on earth are ploughmen, and they get 18*d.* a day.

